# Efficient Synthesis of Norbuprenorphines Coupled with Enkephalins and Investigation of Their Permeability

**DOI:** 10.22037/ijpr.2019.14712.12602

**Published:** 2019

**Authors:** Saeed Balalaie, Morteza Malakoutikhah, Meritxell Teixidó, Vaezeh Fathi Vavsari, Ernest Giralt, Yaghoub Haghighatnia, Fatima Hamdan, Armin Arabanian

**Affiliations:** a *Peptide Chemistry Research Center, K. N. Toosi University of Technology, Tehran, Iran.*; b *Medical Biology Research Center, Kermanshah University of Medical Sciences, Kermanshah, Iran.*; c *Institute for Research in Biomedicine (IRB Barcelona), Barcelona Institute of Science and Technology (BIST), Baldiri Reixac 10, 08028 Barcelona, Spain.*

**Keywords:** Enkephalin, Permeability, Norbuprenorphine, Membrane, Coupling, Solid phase peptide synthesis

## Abstract

An efficient approach for the synthesis of norbuprenorphin derivatives through coupling of enkephalins and norbuprenorphine intermediates is described. Norbuprenorphine derivative was synthesized from thebaine and then, its reaction with succinic acid and phthalic acid was also studied. Meanwhile, the synthesis of enkephalins was done using solid phase peptide synthesis approach. Furthermore, after cleavage of the peptide from the surface of the resin, the coupling of enkephalins with norbuprenorphine derivative was done using TBTU as a coupling reagent then the derivatives were purified using preparative high-pressure liquid chromatography and their structures were confirmed using high-resolution mass spectrometry data. Later, their permeability across membranes was investigated. After PAMPA studies, it was found that the permeability of all norbuprenorphin-enkephalin derivatives was increased; however, succinic and phthalic acid derivatives showed higher permeability than norbuprenorphine-Leu-enkephalin.

## Introduction

The ability to design small molecules possessing the key structural and functional elements of biologically active peptides is a central goal in drug discovery (1,2). Enkephalins **2 **,**1** are endogenous peptides with opioid activity. They are highly flexible pentapeptides that can exist in numerous conformations (3). Structure–activity relationship (SAR) studies showed that additional amide bonds and also existence of aromatic groups enhanced receptor affinity and also the lipophilicity of the synthesized peptides (4-9). 

**Figure 1:**
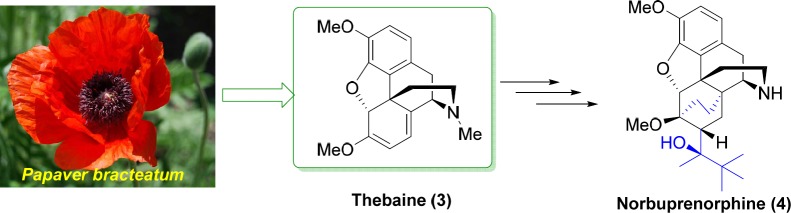
The origin of thebaine and its further use for synthesis of norbuprenorphine

As shown in Figure 1, thebaine **3** is the main opiate alkaloid extracted from *Papaver bracteatum* (Iranian poppy) (10, 11). Thebaine **3** is used for preparation of a variety of medicines including naltrexone, oxycodone, nalbuphine, naloxone, oxymorphone, buprenorphine, and etorphine (12-14). Buprenorphine, as a semisynthetic opioid, is a mixed partial agonist opioid receptor modulator which is applied in lower dosages to control moderate acute and chronic pains, and in higher dosages to treat opioid addiction (15). Norbuprenorphine **4** (Figure 1) is the main active metabolite of the buprenorphine with very limited blood-brain-barrier penetration (15, 16). Continuing our previous studies (17-21), in this research we report the synthesis of norbuprenorphines coupled with Met- and Leu-enkephalins enkephalins neuropeptides. The purpose of this study is to investigate the permeability of the norbuprenorphine coupled to enkephalins across the blood-brain-barrier compared to 

enkephalins.

## Experimental

*General*

The reagents were purchased from Merck and were used without purification unless otherwise stated. Flash column chromatography was carried out using silica Gel 60 (particle size 0.04–0.06 mm/230–400 mesh). The Fourier transform infrared spectroscopy (FT-IR) spectra were recorded on an ABB FT-IR (FTLA 2000) on KBr plate. High-resolution mass spectrometry (HRMS) was recorded on a Mass-ESI-POS (Apex Qe-FT-ICR instrument). Nuclear magnetic resonance (NMR) spectra were recorded at BRUKER AVANCE DRX-500 (for ^1^H-NMR) and DRX-125 MHz (for ^13^C-NMR) in DMSO-d_6_ using tetramethylsilane (TMS) as internal standard. For purification and investigation of purified compounds, high-performance liquid chromatography (HPLC) was performed using a preparative HPLC pump 1800 (KNAUER). Acetonitrile and water with a ratio of 70:30, respectively, in the presence of NaH_2_PO_4_ (10 mM) were used as eluent of HPLC. A Freeze-dryer (CHRIST) was used for drying the products. All synthesized compounds, including intermediates and products, were analyzed by FT-IR, ^1^H-NMR, ^13^C-NMR, HPLC, and HRMS.

*Synthetic procedures*

*General procedure for the preparation of Boc-Tyr (t-Bu)-Gly-Gly-Phe-Met-OH*

The reaction was done using 2-CTC resin (1 g) following the standard Fmoc/*t-*Bu strategy. Fmoc Met-OH (0.74 g, 2 mmol) was attached to 2-Chlorotrityl resins (2-CTC) with N,N-Diisopropylethylamine (DIPEA) (1.4 mL, 8 mmol) in anhydrous dichloromethane (DCM): dimethylformamide (DMF) (10 mL, 1:1) during 2 h at room temperature. After filtration, the remaining trityl chloride groups were capped using a solution of DCM/MeOH/DIPEA (17:2:1, 120 mL) for 30 min in three portions. Then, it was washed with DCM (5 mL × 1), DMF (5 mL × 4), and MeOH (5 mL × 5). The loading capacity of the resin was determined by weight after drying under vacuum. The resin-bound Fmoc-amino acid was washed with DMF (3 × 5 mL) and treated with 25% piperidine in DMF (13 mL) for 30 min and the resin was then washed with DMF (3 × 5 mL). In this step, a solution of Fmoc-Phe-OH (0.77 g, 2 mmol), TBTU (0.64 g, 2 mmol), and DIPEA (0.8 mL, 4.7 mmol) in 6.5 mL DMF was added to the resin-Met-OH and shaken for 1 h at ambient temperature. When the coupling was completed, the resin was washed with DMF (4 × 5 mL). 

The coupling route was repeated for other amino acids of their sequences. In all cases, the Kaiser Test was applied in order to detect the presence or absence of free primary amino groups. Fmoc was determined using UV spectroscopy method. By coupling reactions, the resin was also washed with DMF (4 × 5 mL). The peptide was cleaved from resin in the presence of TFA (1%) in DCM (70 mL) while neutralized with pyridine (4%) in MeOH (12 mL). The solvent was removed under reduced pressure and the peptide precipitated in water. The crude peptide was filtered and dried under the reduced pressure at 40 °C (22-24). 

*General procedure for the preparation of Boc-Tyr(t-Bu)-Gly-Gly-Phe-Leu-OH*

It was synthesized with the same procedure to the Boc-Tyr(t-Bu)-Gly-Gly-Phe-Met-OH except in the first step, i.e. Fmoc-Leu-OH (0.71 g, 2 mmol) was used to attach the 2-CTC resin. 

*Synthesis of norbuprenorphine* **4**

Norbuprenorphine **4** was synthesized from thebaine **3** through five reaction steps (Scheme 2). Thebaine **3** (1 mol, 311 g) was firstly reacted with methyl vinyl ketone **5** (1.35 mol, 95 g) in toluene 80 °C for 4-8 h. 

The obtained compound **6** was then converted to compound **7** by catalytic hydrogenation (Pd/C in ethanol). The reaction followed by addition of *t*-BuLi in dry toluene and ether. Compound **8** reacted with cyanogen bromide in chloroform under reflux condition for 5 h, and then it was treated with KOH in diethylene glycol as solvent at 170 °C for 2 h to gain the product **4 **(25). 

*Synthesis of succinic acid derivative of norbuprenorphine* **10**

Norbuprenorphine **4** (0.2 mmol, 850 mg) and succinic anhydride **9** (3 mmol, 300 mg) were stirred in EtOAc (10 mL) at room temperature for 12 h. The mixture was then kept for 2 h at 4 °C and after this time, the precipitate was filtered and dried (820 mg, 78%) (26).

*Synthesis of phthalic acid derivative of norbuprenorphine* **12**

Norbuprenorphine **4** (0.2 mmol, 850 mg) and phthalic anhydride **11** (3 mmol, 444 mg) were stirred in EtOAc (10 mL) at room temperature for 12 h. The mixture was then kept for 2 h at 4 °C and after this time, the precipitate was filtered and dried (992 mg, 86%) (26).

*General procedure for the preparation of norbuprenorphine-enkephalins* **14, 15, 17, 18**

For this aim, Met-enkephalin (146 mg, 0.2 mmol) or Leu-enkephaline (142 mg, 0.2 mmol) was added to a mixture of norbuprenorphine **4** (85 mg, 0.2 mmol), N,N,N′,N′-Tetramethyl-O-(benzotriazol-1-yl) uronium tetrafluoroborate (TBTU) (70 mg, 0.22 mmol), Hydroxybenzotriazole (HOBT) (27 mg, 0.2 mmol), and DIPEA (77 mg, 0.6 mmol) in EtOAc (6 mL) as solvent and the reaction was started at room temperature for 20 h. The reaction progress was monitored by TLC. When the reaction was completed, the work-up procedure was accomplished by dissolving the mixture in EtOAc (5 mL) and washing with citric acid (3×8 mL, 20%), solution of sodium bicarbonate (3×10 mL, 3%), and distilled water (10 mL). After decantation, EtOAc was evaporated and the pure product was obtained. In order to deprotecting the product, it was dissolved in a mixture of TFA/TES/H_2_O (4.4 mL) in a ratio of 94:1:5 and stirred well at ambient temperature for 2 h. Then, the volume of mixture was decreased to 1 mL and Et_2_O (15 mL) added to it. After 2 h, the precipitate was filtered, washed with a minimum amount of ether and dried under reduced pressure at 40 °C. The products were purified using HPLC.

*General procedure for the preparation of succinic acid derivative of norbuprenorphine-Leu-enkephalin* **23**

Succinic acid derivative of norbuprenorphine **10 **(1.05 mg, 2 mmol) was added to [Leu(Boc)-enkephalin-resin] in the presence of TBTU (640 mg, 2 mmol) and DIPEA (0.8 mL, 4.7 mmol) in DMF (6.5 mL) and well mixed at room temperature. After the completion of the coupling reaction , monitored by Kaiser Test, it was filtered and washed well with DMF and DCM. In order to separate resin from the peptide-enkephalin, TFA (40 mL, 1%) was added to the dissolved resin-peptide-enkephaline **19** in DCM in some portions. Then, the separated peptide from resin was neutralized with pyridine (12 mL, 4%) in MeOH. Following, the solvent was approximately evaporated and water (7 mL) was added to it. After 1 h, the precipitate was filtered and dried under the reduced pressure at 40 °C. To deprotect norbuprenorphine moiety, the peptide **21** (216 mg) was dissolved in a mixture of TFA/TES/H_2_O (4.4 mL) in a ratio of 94:1:5 and stirred well at ambient temperature for 2 h. Then, the volume of mixture was decreased to 1 mL and Et_2_O (15 mL) added to it. After 2 h, the precipitate was filtered, washed with a minimum amount of ether and dried under reduced pressure at 40 °C.

**Figure 2:**
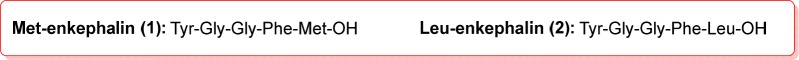
Amino acid sequence of Met- and Leu- enkephalins

**Figure 3:**
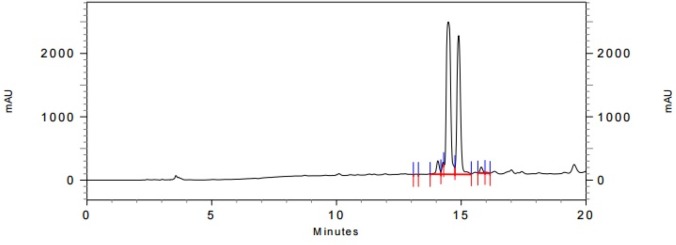
HPLC chromatogram of crude norbuprenorphine and Leu-enkephalin

**Figure 4:**
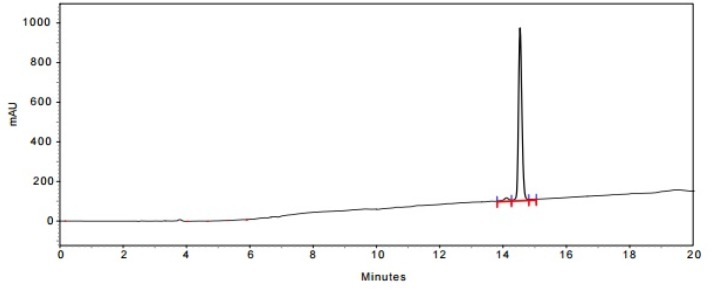
The expanded initial peak of product **14** in HPLC analysis

**Figure 5:**
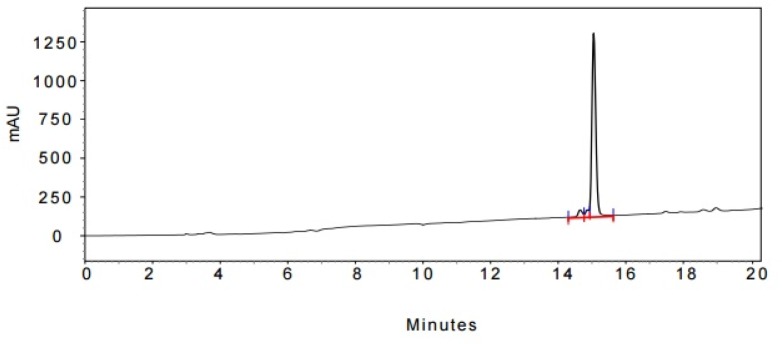
The expanded initial peak of product **15** in HPLC analysis

**Scheme 1:**
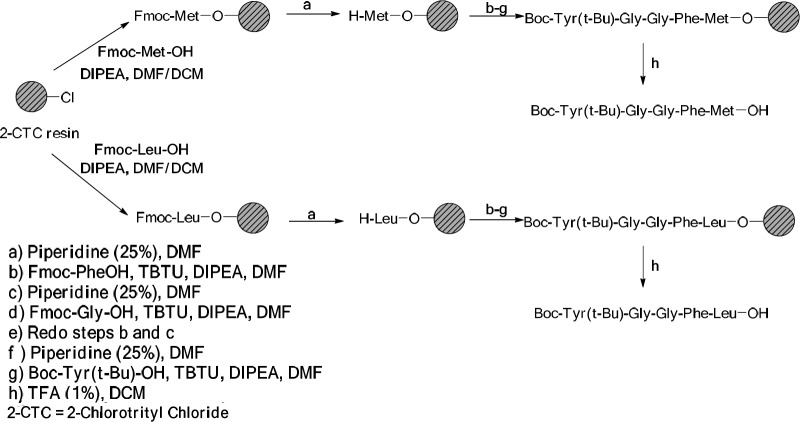
The procedure for the preparation of Met- and Leu-enkephalin

**Scheme 2:**
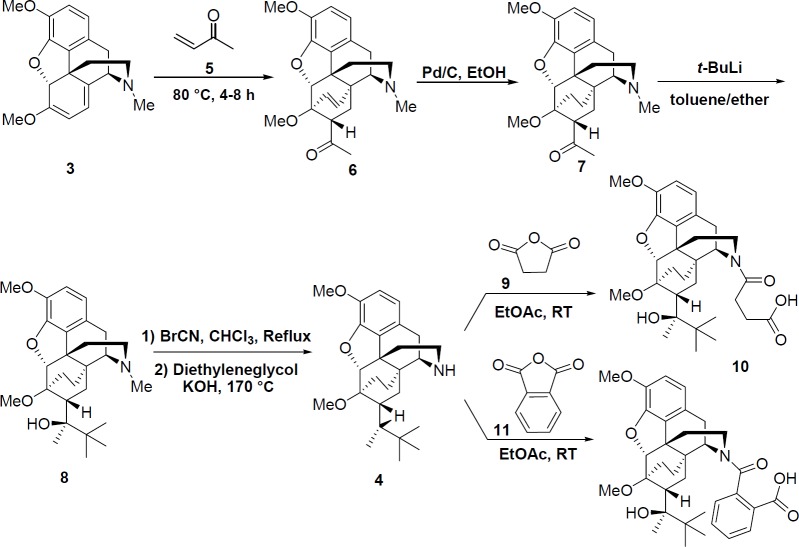
Synthesis of norbuprenorphine and its derivatives

**Scheme 3:**
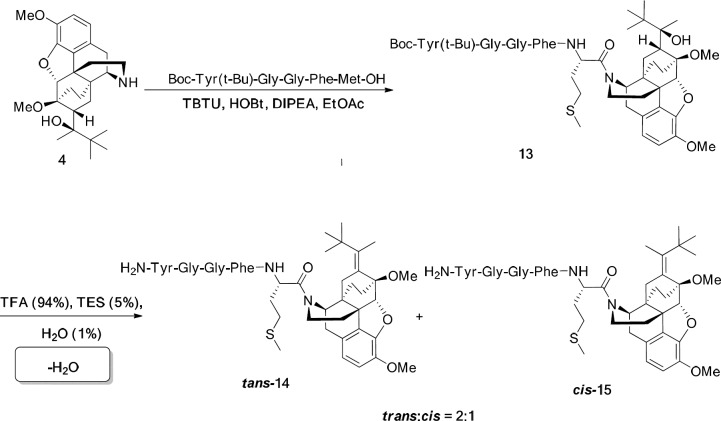
Synthesis of Met-enkephalin-norbuprenorphine

**Scheme 4:**
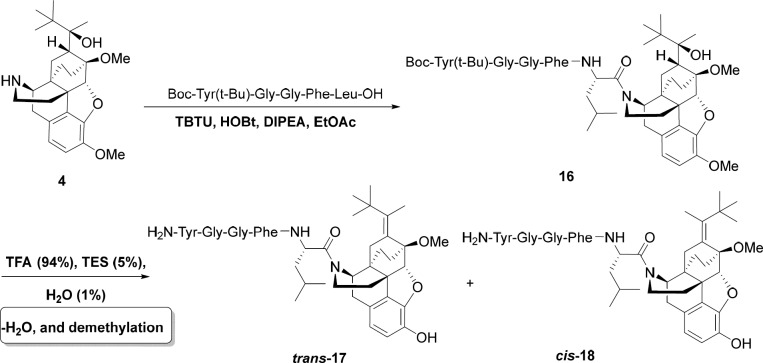
Synthesis of Leu-enkephalin-norbuprenorphine

**Scheme 5:**
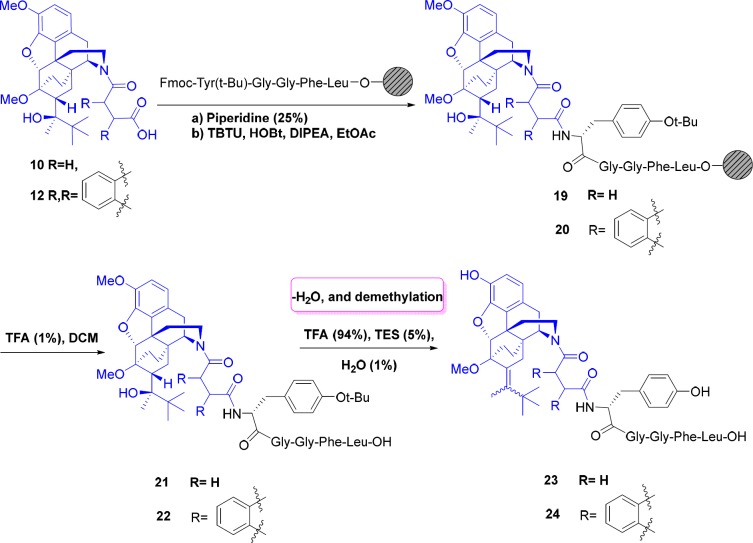
Synthesis of Leu-enkephalin-norbuprenorphine derivatives with linkers

**Table 1:** Percentage of transport after 4 h and effective permeability (Pe) Found in the PAMPA for enkephalins

	**Low permeability**	**Medium permeability**	**High permeability**
**Papp (cm/s)**	**-6** **< 0.1 x 10 cm/s**	**-6 -6** **0.1 x 10 ≤ Papp < 1x 10 cm/s**	**-6** **≥ 1 x 10 cm/s**
**Compound**	**%Membrane retention**	**P** **e ** **× 10 (cm/s)** **-6**	**% PAMPA Transport after 4h**
Standard Met-enkephalin	0	0.09	0.2%
Compound **14**	0	0.77	1.6%
Compound **15**	0	1.42	2.9%
Standard Leu-enkephalin	0	0.38	0.8%
Compound **17**	10.3%	3.46	6.8%
Compound **18**	30.9%	2.13	4.3%
Compound **23**	0	0.63	1.2%
Compound **24**	0	0.92	1.9%
Propanolol	21.8%	5.23	9.9%

*General procedure for the preparation of phthalic acid derivative of norbuprenorphine-Leu-enkephalin* **24**

The procedure of this synthesis was completely the same as the method of succinic acid derivative of norbuprenorphine-Leu-enkephalin **23**.

*Procedure for Parallel Artificial Membrane Permeability Assay (PAMPA) evaluation of norbuprenorphine-enkephalin derivatives*

The PAMPA was used to determine the capacity of compounds to cross a model of the BBB by passive diffusion. The effective permeability of the compounds was measured at an initial concentration of 200 μM. The buffer solution was prepared from a concentrated one commercialized by PION, and the manufacturer’s instructions were followed. The pH was adjusted to 7.4 using a 0.5 M NaOH solution. The compound of interest was dissolved in buffer solution and 1-propanol (20%, cosolvent) to the desired concentration (200 μM). 

The PAMPA sandwich was separated, and the donor well was filled with 195 μL of the compound solution of interest. The acceptor plate was placed into the donor plate, ensuring that the underside of the membrane was in contact with buffer. An amount of 4 μL of the mixture of phospholipids (20 mg/mL) in dodecane was added to the filter of each well, and 160 μL of buffer solution and 40 μL of 1-propanol were added to the each acceptor well. 

The plate was covered and incubated at room temperature in a saturated humidity atmosphere for 4 h agitation at 25 μm of UWL in the GUT-BOX apparatus. Upon the completion of this time, 150 μL/well from the donor plate and 150 μL/well from the acceptor plate were transferred to HPLC vials and an amount of 100 μL for acceptor pools, 5 μL for donor pools and 5 μL for T_0_ samples were injected in a HPLC reverse-phase symmetry C18 column (150 mm × 4.6 mm × 5 μm, 100 Å, Waters). Transport was also confirmed by HPLC-MS and MALDI-TOF spectrometry in order to be sure the compound had kept its integrity. 

The phospholipid mixture used was a porcine polar brain lipid extract. Composition was 12.6% phosphatidylcholine (PC), 33.1% phosphatidylethanolamine (PE), 18.5% phosphatidylserine (PS), 4.1% phosphatidylinositol (PI), 0.8% phosphatidic acid, and 30.9% of the other compounds.

Analysis of PAMPA assay was done using HPLC. Donor and Acceptor pools of each sample were analyzed by triplicate. Thus, a series of HPLC analysis for each sample were performed injecting those quantities and averaging both T_0_ analysis using a 8 minute 0 to 100 gradient (H_2_O, MeCN) in a Symmetry C18 column (150 mm x 4.6 mm x 5 μm, 100 Å, Waters): Integration of area peaks was carried out, normalization of areas was done and calculations of transport (%) and permeability were performed (27, 28).

## Results and Discussion

Our goal is to conjunct norbuprenorphine with Met- and Leu- enkephalin peptides. To access this goal, the conjunction was made through amidation between the free carboxylic acid of the Met- and Leu-enkephalin and the amine group of norbuprenorphine using a suitable coupling reagent. 

*Synthesis of enkephalins*

Solid Phase Peptide Synthesis (SPPS) method was used to synthesize Met- and Leu-enkephalins. 

Scheme 1 shows the process for the synthesis of the peptide moieties (Met- and Leu-enkephalin).

*Synthesis of norbuprenorphine and its derivatives*

In the next stage (Scheme 2), norbuprenorphine **4** was synthesized from thebaine **3** as the starting material. Accordingly, thebaine **3** was reacted with methyl vinyl ketone **5** followed by hydrogenation process in the presence of Pd/C. This solution was gradually added to the organolithium reagent (*t*-BuLi) in toluene, which was then treated with cyanogen bromide. Finally, the norbuprenorphine **4** was obtained in the presence of KOH in ethylene glycol as solvent at 170 °C for 2 h. Furthermore, succinic acid and phthalic acid derivatives of norbuprenorphine (**10 **and** 12**) were synthesized by treating **4** with succinic anhydride **9** and phthalic anhydride **11**, respectively.

*Synthesis of norbuprenorphine-enkephalin derivatives*

In the last stage, norbuprenorphine **4** and its derivatives (**10** and **12**) were coupled to the Met-enkephalin and Leu-enkephalin in the presence of TBTU as coupling reagent. In this regards, norbuprenorphine **4** was coupled with the carboxylic acid moiety of Boc-protected Met-enkephalin (Scheme 3) and/or Leu-enkephalin from its -NH functional group (Scheme 4). Then, the obtained compounds (**13** and **16**) were deprotected in the presence of TFA/TES/H_2_O with a ratio of 94:1:5 and then, purified using preparative HPLC (Figures 2-4). According to the HR-ESI-MS spectrum of the norbuprenorphine-Met-enkephalin (see Scheme 3), since the molecular mass was m/z = 964.47630, it was found that intermediate was dehydrated in the presence of TFA which led to formation of a stable alkene with two conformational isomers of *cis* and *trans *(**14** and **15**). This result is in accordance with the HPLC peaks (Figures 3 and 4) and fragmentation of MS analysis. Furthermore, the same results were obtained for norbuprenorphine-Leu-enkephalin but after deprotection, in addition to dehydration, demethylation also occurred (see compound **17** and **18**).

Next, in order to create enough space between the norbuprenorphine and peptide, and to reduce the steric hindrance, succinic and phthalic acids were applied to form amide bonds as spacers and linkers. For this purpose, after deprotection of amine group on the resin, amide bond was formed between the carboxylic acid functional groups of succinic and phthalic acid derivatives of norbuprenorphine (**10 **and **12**) and NH_2_ group of tyrosine presence in the resin-Leu-enkephalin. These reactions were performed in the presence of coupling agents (TBTU). After completion of the reaction, the peptide was cleaved from the surface of the resin using TFA (1%) and in this case dehydration was not observed. Final deprotection was then accomplished in the presence of TFA/TES/H_2_O (94:5:1). As mentioned before, deprotection of compounds led to dehydration and demethylation and therefore, mixture of *cis* and *trans* isomers of alkenes **23**, **24** were obtained (Scheme 5). The products were purified using preparative HPLC.

*PAMPA evaluation of norbuprenorphine-enkephalin derivatives*

After synthesis of the target molecules, the permeability of these conformations was evaluated. For this aim, an artificial lipid membrane was used. Initially, a given concentration of peptide (200 μM) in the buffer solution and co-solvent (1-propanol, 20%), at pH 7.4 was prepared. Then, it was inserted on the donor well and on another side, the buffer solution and co-solvent (pH 7.4) were placed in the acceptor well. The concentration of peptide in acceptor was then monitored as a function of time. It was found that the concentration was zero at beginning of the process, while it was increased by time as determined by HPLC. 

According to the PAMPA results which are summarized in Table 1, it was found that:

1. Coupling of norbuprenorphine to the peptide increased the permeability of all synthetic norbuprenorphine-enkephalin derivatives. Compound **14 **(0.77×10^-6 ^(cm/s)) and compound** 15 (**1.42×10^-6 ^(cm/s)) showed increased permeability compared to the standard Met-enkephalin (0.09×10^-6 ^(cm/s)).
2. Permeability of Leu-enkephalin and its norbuprenorphine derivatives was greater than Met-enkephalin and its norbuprenorphine derivatives. Compound **17 **(3.46×10^-6 ^(cm/s)) and compound** 18 **(2.13×10^-6 ^(cm/s)) showed increased permeability compared to standard Leu-enkephalin (0.38×10^-6 ^(cm/s)).
3. Succinic and phthalic acid derivatives of norbuprenorphine-Leu-enkephalin (compounds **23, 24**) showed lower permeability than norbuprenorphine-Leu-enkephalin.

## Conclusion

Thebaine **3** (Figure 1) is the main opiate alkaloid extracted from *Papaver bracteatum* (Iranian poppy) which is used for preparation of buprenorphine and norbuprenorphine. Norbuprenorphine **4** is the main active metabolite of the buprenorphine with very limited blood-brain-barrier penetration. In this research, norbuprenorphine was synthesized from thebaine and then, its succinic and phthalic acid derivatives were prepared. Furthermore, in order to increase their permeability to biological barriers through passive diffusion, norbuprenorphine and its derivatives were coupled with the Leu-enkephalin and Met-enkephalin. After PAMPA studies, it was found that the permeability of all norbuprenorphin-enkephalin derivatives was increased; however, succinic and phthalic acid derivatives showed higher permeability than norbuprenorphine-Leu-enkephalin. 
